# Crystallinity assessment of anthropogenic calcites using Raman micro-spectroscopy

**DOI:** 10.1038/s41598-023-39842-8

**Published:** 2023-08-10

**Authors:** Michael B. Toffolo, Iddo Pinkas, Ana Álvaro Gallo, Elisabetta Boaretto

**Affiliations:** 1 Geochronology and Geology Program, Centro Nacional de Investigación sobre la Evolución Humana (CENIEH), Paseo Sierra de Atapuerca 3, 09002 Burgos, Spain; 2 Department of Chemical Research Support, Weizmann Institute of Science, 234 Herzl Street, 7610001 Rehovot, Israel; 3 D-REAMS Radiocarbon Dating Laboratory, Scientific Archaeology Unit, Weizmann Institute of Science, 234 Herzl Street, 7610001 Rehovot, Israel

**Keywords:** Geochemistry, Mineralogy, Infrared spectroscopy, Raman spectroscopy

## Abstract

Anthropogenic calcite is a form of calcium carbonate produced through pyrotechnological activities, and it is the main component of materials such as lime binders and wood ash. This type of calcite is characterized by a significantly lower degree of crystallinity compared with its geogenic counterparts, as a result of different formation processes. The crystallinity of calcite can be determined using infrared spectroscopy in transmission mode, which allows decoupling particle size effect from atomic order and thus effectively distinguish anthropogenic and geogenic calcites. On the contrary, Raman micro-spectroscopy is still in the process of developing a reference framework for the assessment of crystallinity in calcite. Band broadening has been identified as one of the proxies for crystallinity in the Raman spectra of geogenic and anthropogenic calcites. Here we analyze the full width at half maximum of calcite bands in various geogenic and anthropogenic materials, backed against an independent crystallinity reference based on infrared spectroscopy. Results are then used to assess the crystallinity of anthropogenic calcite in archaeological lime binders characterized by different states of preservation, including samples affected by the formation of secondary calcite, and tested on micromorphology thin sections in which lime binders are embedded in sediments.

## Introduction

Calcite is the stable polymorph of calcium carbonate (CaCO_3_) at Earth-surface conditions, and it is commonly found in its geogenic (e.g., limestone, chalk) and biogenic (e.g., foraminifera, mollusks) forms^1,2^. Calcite can also nucleate upon the carbonation of hydrated lime, Ca(OH)_2_, obtained through the thermal decomposition of a CaCO_3_ substrate to quicklime (CaO). The latter is unstable at ambient conditions, and readily reacts with atmospheric humidity and CO_2_ to form again calcite. This process seldom occurs in nature and the resulting calcite is usually associated with pyrotechnological activities, such as the production of lime binders, whereby quicklime is deliberately mixed with water and other components to obtain materials such as lime plaster and mortar^3,4^. These materials are often called anthropogenic calcite^5,6^. Another anthropogenic form is wood ash, which comprises calcite derived from the thermal decomposition of calcium oxalates^7–9^. These formation mechanisms affect crystal properties like domain size and habit, and are conducive to distinct densities of structural defects such as lattice strain and microstrain fluctuations^10,11^. Different densities of structural defects produce different degrees of atomic order or crystallinity, here broadly defined as periodic order in three dimensions at the atomic level. For instance, Iceland spar grows over geologic timescales, producing large and well-defined crystals as a result of three-dimensional periodic order over macroscopic distances. At the other end of the crystallinity spectrum, calcite in plaster nucleates rapidly in nm-sized crystallites that exhibit high concentrations of lattice defects^12–14^.

Changes in crystallinity are usually assessed using X-ray diffraction, the benchmark for the analysis of atomic order. However, variability in the short-range atomic order, as in amorphous calcium carbonate (ACC), and lattice defects in nano-sized anthropogenic calcite, are better characterized at the molecular level through vibrational spectroscopy or by pair distribution function analysis of total X-ray scattering^5,15–17^. Recent advances have shown how distinct densities of structural defects caused by exposure to elevated temperatures and/or rapid nucleation (as in lime binders) affect band broadening and intensity in Fourier transform infrared (FTIR) spectra of calcite^11,18,19^. In particular, the grinding curve method in transmission FTIR provides a swift procedure to assess the degree of atomic order of calcite, regardless of particle size. This is based on the repeated grinding of the same KBr pellet, which allows decoupling the opposite trends that particle-size-dependent optical absorption and atomic order impose on the shape of infrared spectra^12,20^. This method has been applied to research in biomineralization, heritage conservation, and archaeology to distinguish calcites characterized by different degrees of atomic order, which can be linked to specific formation paths as well as diagenetic processes involving the recrystallization of primary anthropogenic calcite crystals^6,21–28^. Similar applications have been developed for the study of crystallinity in aragonite^29,30^ and carbonate hydroxyapatite^31–34^. This method provides information on bulk samples, although FTIR micro-spectroscopy in reflectance mode can effectively distinguish geogenic and anthropogenic calcites based on the position and width of the ν_3_ band (~ 1410 cm^−1^)^24,35^.

On the other hand, Raman spectroscopy has seldom been applied to the characterization of subtle differences in the crystallinity of calcite. Despite the possibility to distinguish polymorphs and the substantial literature on the temperature dependence of linewidths in calcite^36–41^, only a few studies probed the degree of atomic order in CaCO_3_ polymorphs formed by different mechanisms, and mainly based on the contribution of ACC (band at ~ 1075 cm^−1^) to the broadening of the ν_1_ band of calcite and aragonite (1087 cm^−1^)^42–45^. In these works, crystallinity was considered in terms of ACC not yet converted to calcite, rather than defects in the crystal structure of the latter. To explore this issue, a recent study by Calandra et al.^46^ focused on Raman band locations and widths in bulk samples of geogenic and anthropogenic calcites. The authors found that a slight shift in the position of some of the calcite bands may be used to distinguish geogenic calcites from lime mortars, with the aid of a machine learning workflow. In addition, they demonstrated that the full width at half maximum (FWHM) values of the calcite bands show consistent differences between geogenic and anthropogenic calcites, with the former exhibiting narrower bands due to their higher degree of atomic order and larger crystal size. Band shift and broadening were thus proposed as proxies for crystallinity and strain in the analysis of the Raman shift of calcite in cultural heritage materials.

Here we apply these proxies to the analysis of Raman spectra of calcites formed by different mechanisms, and propose a way to assess the crystallinity of archaeological lime binders in petrographic and micromorphology thin sections, which are fundamental tools for the interpretation of the microscopic archaeological record and the conservation of cultural heritage materials^3,4,47,48^. FWHM values are dependent on spectral resolution, which in turn depends on instrumentation, such as spectrometer focal length and density of grooves in the grating. In other words, lower spectral resolution produces larger FWHM values, which should be determined before they can be used in an absolute way to distinguish calcites exhibiting different degrees of crystallinity. Furthermore, changes in the Raman shift of secondary calcite need to be characterized in order to determine the occurrence of diagenetic alterations caused by the recrystallization of primary anthropogenic calcite crystals. By using two instruments with 532 nm laser, 1800 and 900 gr/mm gratings and 800- and 252-mm focal length of the spectrometer, respectively, we monitored band broadening in various types of geogenic and anthropogenic calcites, the latter both experimental and archaeological. In addition to standard materials considered in previous studies, we analyzed wood ash, a form of anthropogenic calcite that is ubiquitous at archaeological sites. The degree of short-range atomic order of these reference materials was determined independently using the FTIR grinding curve method. Results show that the 900 gr/mm grating produces FWHM values that are roughly two times larger than the values obtained with the 1800 gr/mm grating. While absolute values differ, clear-cuts in FWHM values between standards can be used to detect differences in crystallinity. Changes in FWHM in the Raman linewidth allow the identification of anthropogenic calcite altered by diagenesis, in which secondary crystals are characterized by a greater degree of atomic order compared to the parent substrate. These results facilitate the identification of anthropogenic calcite in archaeological sediments and materials at the microscopic scale, especially in thin section, and find application in the preservation of cultural heritage and in the characterization of lime binders and other types of synthetic CaCO_3_.

## Results and discussion

**Figure 1:**
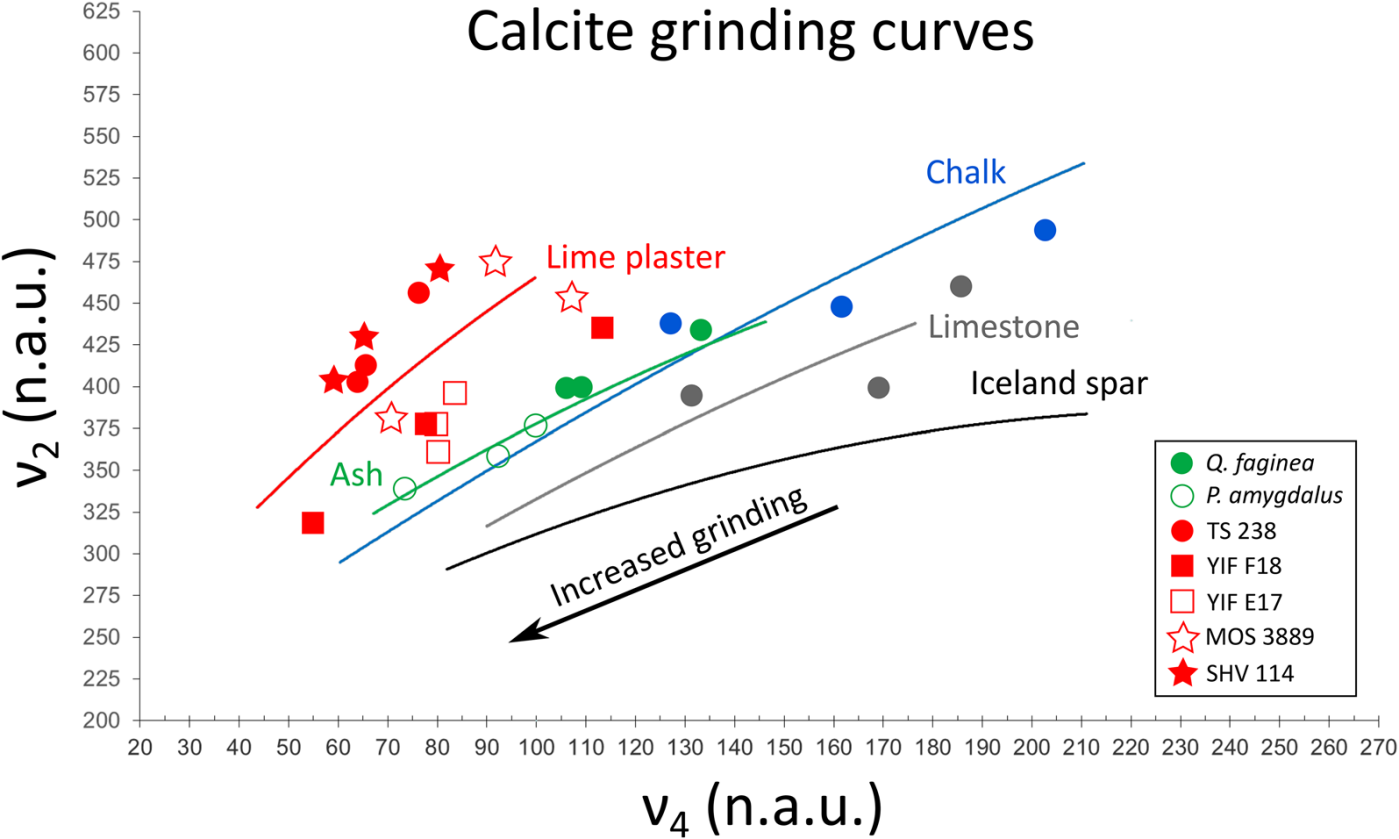
Grinding curve plot showing the locations of the ν_2_ and ν_4_ normalized intensities of representative spectra of calcite standards and archaeological samples (n.a.u.: normalized absorbance units). Blue dots represent Nesher chalk, grey dots represent Sde Boker limestone. Plot reproduced with permission after Regev et al.^20^.

The degree of crystallinity of all materials used in this study was first assessed using the FTIR grinding curve method in transmission mode and published reference curves of calcite standards^20^. We selected a broad range of materials in order to cover the entire crystallinity spectrum between geogenic and anthropogenic extremes, including limestone, chalk, wood ash from two species, experimental lime plaster, and four archaeological lime plasters characterized by different states of preservation. The calcite ν_2_ (875 cm^−1^) and ν_4_ (713 cm^−1^) intensities of chalk and limestone, normalized to the intensity of the respective ν_3_ (1420 cm^−1^)^5,20^, fall close to the relative curves, and a similar pattern can be observed for the ash of *Quercus faginea* (Portuguese oak) and *Prunus amygdalus* (almond), which overlap with the curve of wood ash, and the experimental lime plaster TS 238 that falls immediately above the experimental lime plaster curve (Fig. 1). As shown in earlier studies, the shape of each grinding curve is determined by changes in absorption caused by different particle sizes, whereas the offset of a curve (relative to a simulated ideal curve) depends on the degree of atomic order of crystals^12,20^. Results confirm that geogenic standards are well ordered at the atomic level, whereas anthropogenic calcites are poorly ordered. The archaeological lime plasters exhibit larger variability in their crystallinity. Plaster SHV 114, despite being ~ 1300 years old, exhibits the same degree of atomic order as experimental lime plaster. This has been previously interpreted as a lack of recrystallization in an arid environment (helped also by the relatively young age of the material), given that the collection site is located in the Negev Desert^24^. Calcite in plaster MOS 3889 falls between the curves of experimental lime plaster and wood ash, indicating a certain degree of recrystallization, whereby part of the primary anthropogenic calcite dissolved and reprecipitated as larger and more ordered crystals^30^. The occurrence of aragonite of anthropogenic origin in MOS 3889 is a proxy for overall good preservation of the lime binder, since aragonite is more soluble than calcite^49^. In addition, a previous study showed that the aragonite in this sample is anthropogenic and primary (not recrystallized), based on its degree of atomic order^30^. The normalized intensities of plasters YIF E17 and YIF F18 plot closer to the wood ash curve, due to extensive recrystallization and the precipitation of large secondary calcite crystals^35^ (Fig. 1). Therefore, FTIR results show that the geogenic samples are the most ordered at the atomic level, whereas the experimental plaster and SHV 114 are the least ordered. Experimental wood ashes are slightly less ordered than geogenic standards, and archaeological plasters are characterized by intermediate degrees of crystallinity, with the exception of SHV 114 based on its exceptional state of preservation. These results provided a reference for the calcite crystallinity spectrum.

**Figure 2:**
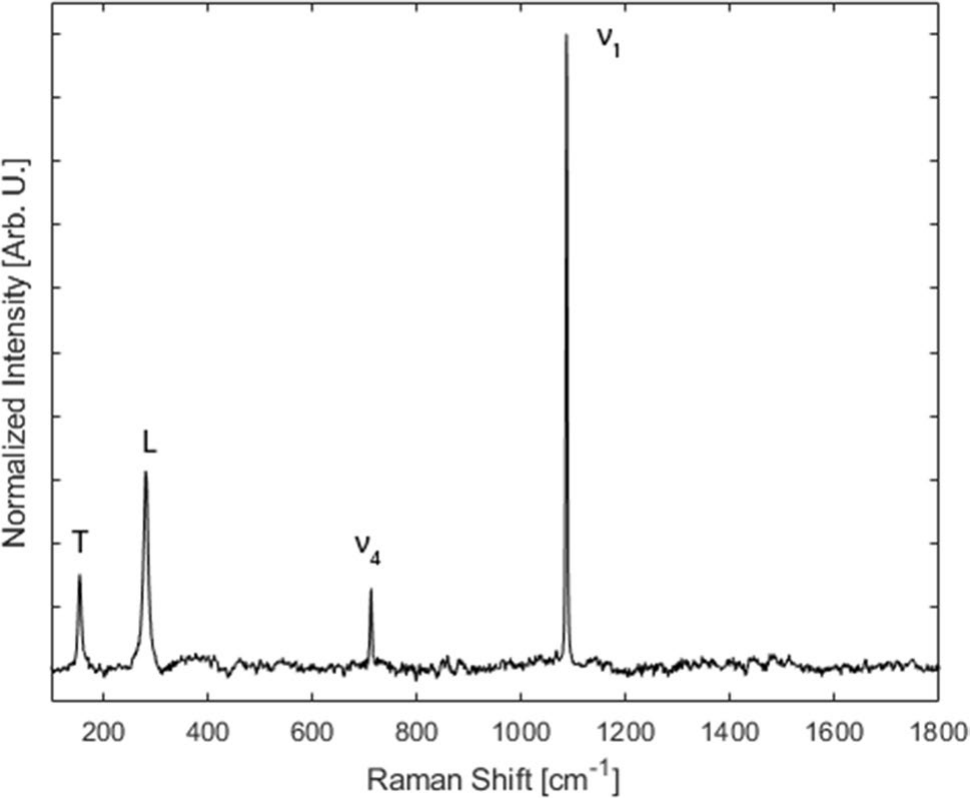
Representative geogenic calcite spectrum showing the location of the bands discussed in the text.

Analyses by Raman micro-spectroscopy using the 1800 gr/mm grating focused on establishing a reference framework for crystallinity in geogenic and anthropogenic standards by measuring band location and FWHM. The Raman bands of calcite in the low-medium spectral range include the translational (T) mode at 155 cm^−1^, the librational (L) mode at 281 cm^−1^, and the ν_4_ in-plane bending at 712 cm^−1^, all of these in the *E*_*g*_ symmetry group; and the ν_1_ symmetric stretching at 1087 cm^−1^, in the *A*_*1g*_ symmetry group^50^ (Fig. 2). Results show that the average shifts of the 155, 281, 712, and 1087 cm^−1^ bands of experimental plaster TS 238 are consistently characterized by slightly lower wavenumbers compared to limestone and chalk, thus confirming previous observations^46^ (Table 1). Therefore, the location of these bands may be used to distinguish geogenic and anthropogenic calcites. Based on this reference, we applied the same proxy to archaeological lime plasters in thin section, with the aim of exploring possible correlations with the FTIR grinding curves in terms of crystallinity. In general, the L, ν_4_, and ν_1_ vibrations confirm that anthropogenic calcites are characterized by lower wavenumbers, whereas the T mode shows no pattern, with plasters YIF E17 and YIF F18 showing higher wavenumbers compared to the geogenic standards (Table 1). However, while geogenic and anthropogenic calcites can be clearly differentiated, no distinction can be made between plasters characterized by different degrees of crystallinity. In addition, considering that values significantly overlap between geogenic and anthropogenic materials, a large number of measurements is required to determine whether a sample of unknown provenience can be assigned to one of the two groups.

**Table 1:** Average Raman shift values in calcite standards and archaeological samples measured with the 1800 gr/mm grating and 800 mm spectrometer focal length.

Sample	T (cm^−1^)	L (cm^−1^)	ν_4_ (cm^−1^)	ν_1_ (cm^−1^)
Chalk	155.2 ± 0.1	281.7 ± 0.1	712.8 ± 0.1	1087
Limestone	155.5 ± 0.2	281.9 ± 0.1	712.7 ± 0.3	1087
YIF F18	156.1 ± 0.7	280.7 ± 0.6	712.2 ± 0.3	1086.7 ± 0.4
YIF E17	156.1 ± 0.8	281.1 ± 0.5	712.1 ± 0.4	1086.4 ± 0.5
MOS 3889	155.9 ± 0.7	281.3 ± 0.5	712.5 ± 0.4	1087 ± 0.2
TS 238	154.9 ± 0.3	280.7 ± 0.2	712.4 ± 0.2	1086.4 ± 0.5
SHV 114	155.5 ± 0.4	280 ± 0.3	712 ± 0.2	1086 ± 0.2

**Figure 3:**
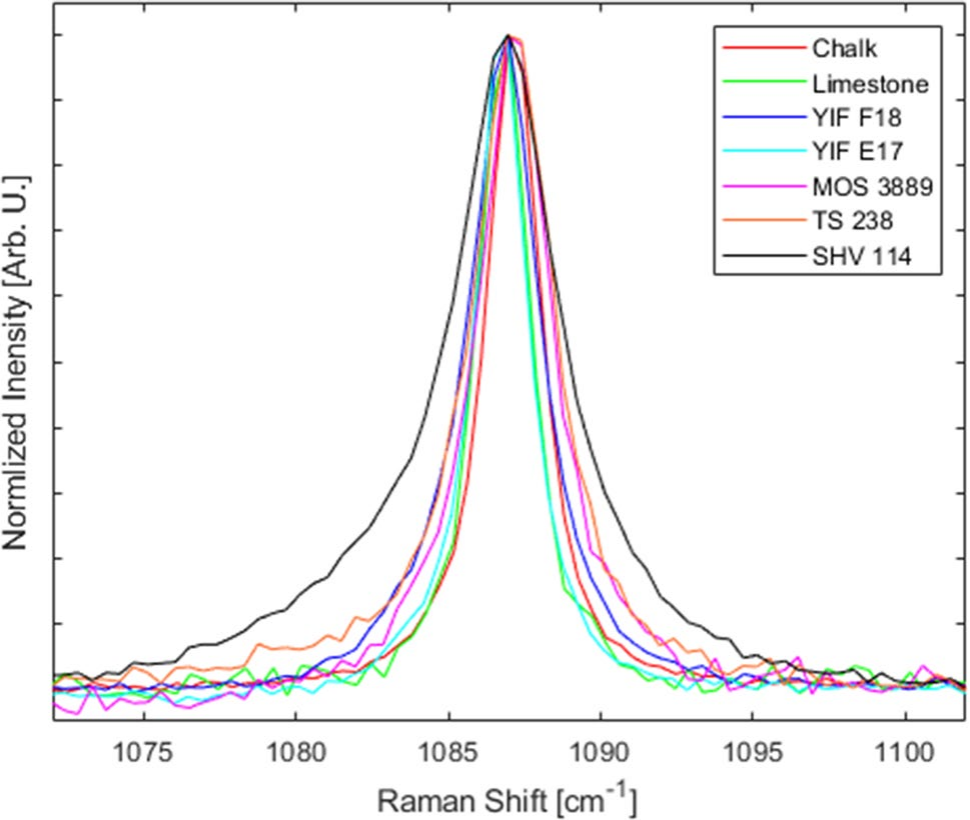
Representative spectra of the calcite samples analyzed in this study, illustrating the differences in FWHM of the ν_1_ vibration between geogenic and anthropogenic materials.

**Figure 4:**
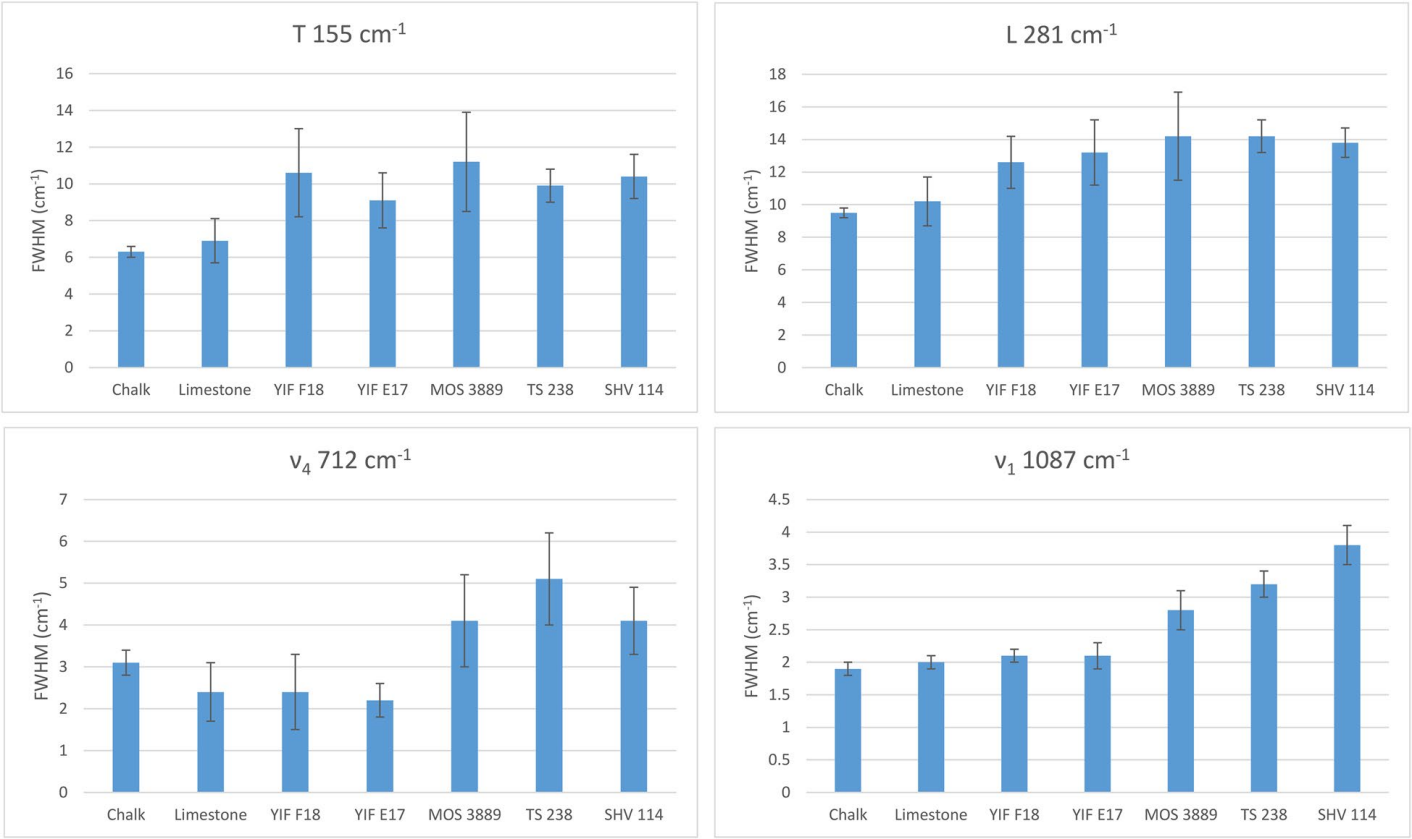
Average FWHM of Raman bands of calcite standards and archaeological samples measured with the 1800 gr/mm grating and 800 mm spectrometer focal length.

The analysis of Raman linewidths provided further insights into the crystallinity of the various specimens within the anthropogenic group. Geogenic calcites exhibit smaller FWHM values compared to experimental lime plaster, and therefore could be used as a proxy for crystallinity provided that a large number of measurements is carried out, considering the large standard deviations in most bands (Table 2 and Figs. 3, 4). This trend is caused by the higher degree of atomic order in geogenic crystals, and it has been observed also in carbonate hydroxyapatite, where the 961 cm^−1^ band (ν_1_) of the phosphate functional group is broader in fresh bone, which is characterized by small and poorly ordered crystals, and narrower in archaeological bones altered by diagenesis, in which crystals are larger and more ordered^51^. Although it has not yet been shown that Raman micro-spectroscopy can decouple particle size effect from crystallinity in calcite, we can assume that both the small particle size and poor degree of atomic order of calcite crystals in experimental lime plaster contribute to the broadening of bands, whereas the relatively large and well-ordered crystals in chalk and limestone favor band narrowing. A similar pattern was found in biogenic aragonite^42^. However, by analyzing the FWHM of calcite in archaeological lime plasters, we found that the 712 cm^−1^ band shows no clear pattern linked to crystallinity, since the YIF samples exhibit narrower bands compared to the geogenic standards (Table 2 and Fig. 4). This is probably caused by the much higher level of noise that affects this band. Further distinctions within the anthropogenic calcite group can be made based on the FWHM of the 1087 cm^−1^ band, which exhibits a FWHM pattern consistent with the degree of atomic order observed using the FTIR grinding curves. Chalk and limestone exhibit the smallest FWHM values, plasters TS 238 and SHV 114 the largest. Plaster MOS 3889 shows an intermediate value between geogenic and anthropogenic calcite, although closer to plaster TS 238. The heavily recrystallized YIF plasters exhibit FWHM values closer to the geogenic standards (Fig. 4). These trends are consistent with the fact that the ν_1_ reflects the internal vibration of CO_3_^2−^ functional groups, rather than the entire calcite unit cell as is the case of the T and L vibrations. The results obtained from the 1087 cm^−1^ band are also characterized by a smaller standard deviation, thus allowing distinctions in crystallinity based on a smaller number of measurements compared to the FWHM of other bands, or to band locations. Therefore, it appears that the ν_1_ provides the best clear-cut between samples characterized by different degrees of structural order. In addition, considering the overlap between geogenic standards and YIF recrystallized plasters, distinction between the two groups can be aided by looking at the FWHM of the L mode, which presents less overlap, in combination with the FWHM of the ν_1_.

**Table 2:** Average FWHM of Raman bands of calcite standards and archaeological samples measured with the 1800 gr/mm grating and 800 mm spectrometer focal length.

Sample	T FWHM (cm^−1^)	L FWHM (cm^−1^)	ν_4_ FWHM (cm^−1^)	ν_1_ FWHM (cm^−1^)
Chalk	6.3 ± 0.3	9.5 ± 0.3	3.1 ± 0.3	1.9 ± 0.1
Limestone	6.9 ± 1.2	10.2 ± 1.5	2.4 ± 0.7	2 ± 0.1
YIF F18	10.6 ± 2.4	12.6 ± 1.6	2.4 ± 0.9	2.1 ± 0.1
YIF E17	9.1 ± 1.5	13.2 ± 2	2.2 ± 0.4	2.1 ± 0.2
MOS 3889	11.2 ± 2.7	14.2 ± 2.7	4.1 ± 1.1	2.8 ± 0.3
TS 238	9.9 ± 0.9	14.2 ± 1	5.1 ± 1.1	3.2 ± 0.2
SHV 114	10.4 ± 1.2	13.8 ± 0.8	4.1 ± 0.8	3.8 ± 0.3

**Figure 5:**
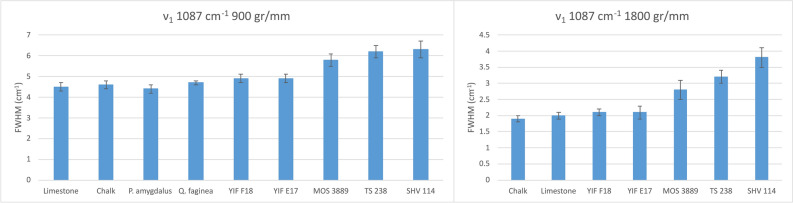
Average FWHM of the Raman ν_1_ band in calcite standards and archaeological samples measured with the 900 and 1800 gr/mm gratings (252 and 800 mm spectrometer focal length, respectively).

Given that the 1087 cm^−1^ band is better suited as proxy for crystallinity, we monitored changes in its FWHM in the different calcite standards using the 900 gr/mm grating and a spectrometer with lower focal length, which provide lower spectral resolution and thus produce broader bands and greater FWHM values. The same pattern was observed, with limestone and chalk showing the smallest FWHM average values, the experimental plaster showing the largest values together with SHV 114, and the archaeological plasters showing intermediate values, with MOS 3889 less crystalline than the YIF plasters. The wood ash of both *Q. faginea* and *P. amygdalus* is characterized by average FWHM values that overlap with limestone and chalk, similar to the overlap between wood ash and chalk in the FTIR grinding curve plot (Fig. 5). The average FWHM values are roughly two times those calculated using the 1800 gr/mm grating, which is twice as much dense (Table 3). While this cannot be used as a conversion factor, it confirms that the distinction between calcites formed by different mechanisms based on the ν_1_ broadening is consistent regardless of instrument settings. This behavior can be used to propose thresholds between different degrees of crystallinity. With a 900 gr/mm grating, FWHM values up to 4.6 cm^−1^ reflect geogenic materials, values between 4.6 and 5 wood ash and recrystallized plaster, and above 5 they can be assigned to well-preserved plaster. Using the 1800 gr/mm grating, the same thresholds are set at 2 and 2.5 cm^−1^, respectively. The FWHM of the L mode can aid the distinction between limestone and heavily recrystallized plasters. If gratings of different density are used, it is advisable to develop a dedicated reference of calcite standards, which can later be applied to samples of unknown crystallinity or provenience. Geogenic standards and wood ash cannot be effectively distinguished using the 900 gr/mm grating. However, if the calcite sample comes from an archaeological site where no geogenic calcite is present in the bedrock or sediments (e.g., substrates made of sandstone, mudstone, igneous rocks, and their breakdown products), FWHM values up to 4.7 cm^−1^ may be assigned to wood ash.

**Table 3:** Average Raman ν_1_ FWHM of the calcite standards and archaeological samples measured with the 900 and 1800 gr/mm gratings (252 and 800 mm spectrometer focal length, respectively).

Sample	ν_1_ FWHM (cm^−1^), 900 gr/mm grating	ν_1_ FWHM (cm^−1^), 1800 gr/mm grating
Limestone	4.5 ± 0.2	2 ± 0.1
Chalk	4.6 ± 0.2	1.9 ± 0.1
*P. amygdalus* ash	4.4 ± 0.2	Not measured
*Q. faginea* ash	4.7 ± 0.1	Not measured
YIF F18	4.9 ± 0.2	2.1 ± 0.1
YIF E17	4.9 ± 0.2	2.1 ± 0.2
MOS 3889	5.8 ± 0.3	2.8 ± 0.3
TS 238	6.2 ± 0.3	3.2 ± 0.2
SHV 114	6.3 ± 0.4	3.8 ± 0.3

**Figure 6:**
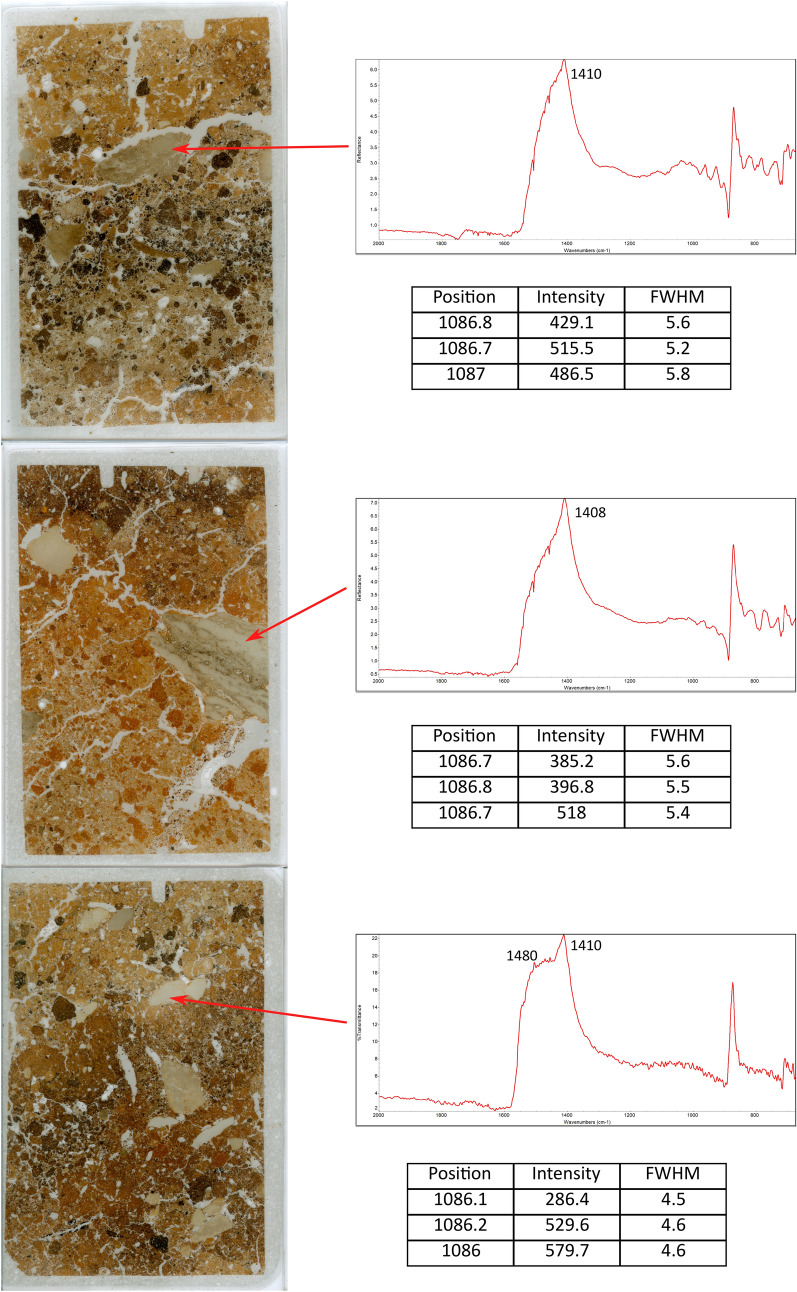
Scans of micromorphology thin sections from Nesher-Ramla quarry, showing the results of FTIR and Raman micro-spectroscopy (900 gr/mm grating, 252 mm spectrometer focal length) of calcite fragments. FTIR spectra show the ν_3_ of calcite, whereas tables illustrate three measurements of the Raman ν_1_ of calcite. Band position and FWHM are expressed in cm^−1^, intensity in absorbance units; short side of thin sections: 5 cm.

The proposed thresholds were applied to the analysis of micromorphology thin sections of sediments from the Pre-Pottery Neolithic B site of Nesher-Ramla quarry (Israel), a sunken lime kiln located at the bottom of a shallow sinkhole and dated to ~ 10,400 years ago^52^. Sediments are rich in clay minerals and fine-grained calcite from the chalk and limestone bedrock, but contain also heated clay minerals from the pyrotechnological activities linked to quicklime production and large fragments of lime plaster. The latter were identified in a previous study using FTIR micro-spectroscopy in reflectance mode based on the position and width of the ν_3_ of calcite, which are known to differ between anthropogenic and geogenic calcites: narrow band at ~ 1410 cm^−1^ in lime plaster and an additional broad band at ~ 1480 cm^−1^ in chalk and micritic limestone^35,53^. The combined results of FTIR and Raman micro-spectroscopy (900 gr/mm grating and 252 mm spectrometer focal length) on the large calcite fragments visible in thin section consistently show that they are characterized by a poor degree of atomic order, typical of experimental lime plaster, with FWHM values of the Raman ν_1_ of calcite above 5 cm^−1^. On the contrary, the few unheated limestone fragments in the kiln sedimentary deposits exhibit FWHM values that fall beneath the geogenic threshold (Fig. 6).

The results presented in this study confirm that Raman band broadening may be used as proxy for crystallinity to distinguish geogenic and anthropogenic calcites. More specifically, the FWHM of the L and ν_1_ vibrations provide the best clear-cuts between standard materials, and allow the identification of recrystallized lime plaster, in which a significant amount of the parent material has dissolved and reprecipitated to produce crystals that are larger and more ordered at the atomic level. These proxies are verified regardless of instrument setup, as we demonstrated with the 900 and 1800 gr/mm gratings, although different grating densities require dedicated analyses of calcite standards in order to use the FWHM values in an absolute manner to distinguish between degrees of crystallinity. Besides the analysis of bulk samples, we showed that the FWHM values are readily applicable to the analysis of petrographic and micromorphology thin sections, thus aiding the crystallinity assessment of calcite in a spatially resolved layout, and at higher resolution compared to routine FTIR micro-spectroscopy in reflectance mode, which is generally limited to 20 µm spatial resolution. Therefore, the application of Raman micro-spectroscopy to the analysis of calcite provides an alternative to FTIR spectroscopy, and can complement it in the analysis of small regions of interest in thin section. Similar to FTIR micro-spectroscopy, mapping of thin sections using Raman imaging may provide large-scale assessments of calcite crystallinity^24,51,54^. This is a fundamental prerequisite for accurate radiocarbon dating of lime binders^55^ and geogenic calcites sampled through laser ablation^56^, for the characterization of ancient lime binders and their self-healing properties^54^, and for the monitoring of the carbonation process of synthetic binders^44,57^.

## Methods

### Samples

Analyses were conducted on standard reference calcites formed by different mechanisms, which are known to exhibit different degrees of short-range atomic order^20^. Geogenic calcite includes limestone from Sde Boker and chalk from Nesher Ramla (both located in Israel). These materials are characterized by well-ordered crystals. Anthropogenic calcite includes an experimental lime plaster and wood ash from two species, which are characterized by poor atomic order. Plaster TS 238 was made in 2008 by decomposing at 800 °C *nari* (recrystallized chalk) from Israel, and by mixing the resulting quicklime with excess water^20^. Hydrated lime was left to react with the atmosphere at room conditions (not monitored). Ash powder was obtained by heating the debarked wood of *Quercus faginea* and *Prunus amygdalus* to 550ºC in a muffle oven for 6 h. Archaeological lime plasters fall within the anthropogenic calcite category, but exhibit different degrees of structural order caused by recrystallization over time. Plaster SHV 114 was collected from the wall of a Byzantine church at Shivta (Israel), dated to the 6^th^–7^th^ century CE^24,58^. Plaster MOS 3889 was collected from a floor at the early Pre-Pottery Neolithic B (PPNB) site of Motza in Israel, dated to ~ 10,500 years ago, and characterized by mild recrystallization based on the occurrence of highly soluble aragonite^30,59,60^. Plasters YIF E17 and YIF F18 were collected from floors at the middle-late PPNB site of Yiftahel in Israel, dated to ~ 9,000 years ago, and are affected by extensive recrystallization including the precipitation of sparite^35,61^.

### FTIR spectroscopy

All samples were analyzed by FTIR in transmission mode to compare them to calcite standards obtained with the grinding curve method, which allows separating atomic order from particle size effect by monitoring the intensity of the ν_2_ and ν_4_ absorptions of calcite (normalized to its ν_3_ absorption^5^) upon repeated grinding of the same KBr pellet, and thus determine their degree of short-range atomic order^12,20^. About 5 mg of each sample were powdered using an agate mortar and pestle and mixed with 40 mg of FTIR-grade KBr. The mixture was pressed into a 7-mm pellet with a hand press, and analyzed in transmission mode using a Thermo Scientific Nicolet iS5 spectrometer at 4 cm^−1^ spectral resolution in 32 scans, in the 4000–400 cm^−1^ spectral range. Spectra were processed using OMNIC v. 9.13 and Macros Basic v. 10, and the normalized ν_2_ and ν_4_ absorptions of calcite were compared with published grinding curves of calcite standards^20^.

### Raman micro-spectroscopy

Samples were analyzed with Raman micro-spectroscopy to investigate the variability in location and linewidth of the calcite bands in relation to their formation mechanism. Considering that archaeological lime plasters are heterogeneous materials where calcite is usually mixed with other components^4^, Raman micro-spectroscopy was performed on petrographic thin sections (30 µm thick) to facilitate the selection of regions of interest rich in calcite^62^. All other samples were analyzed in bulk form by selecting sufficiently flat surfaces. Raman measurements were conducted using a Horiba LabRAM HR Evolution instrument equipped with a 532 nm laser and 1800 gr/mm grating, and 800 mm spectrometer focal length. The system incorporates an open confocal microscope (Olympus BXFM) with a spatial resolution of 0.3 μm when using the 50 × NA = 0.5 objective and 532 nm laser. The geogenic standards were analyzed at 50 different points, TS238 at 110 points, MOS 3889 at 221 points, YIF E17 at 331 points, YIF F18 at 375 points, and SHV 114 at 316 points. Spectra were processed with Horiba LabSpec 6 using the Gaussian/Lorentzian function and polynomial baseline to determine the location, intensity, and FWHM of the calcite bands. Additional analyses (at least 50 points per sample) were performed using a Thermo Scientific DXR Raman micro-spectrometer equipped with a 532 nm laser and 900 gr/mm grating, and 252 mm spectrometer focal length, to monitor changes in the FWHM of the 1087 cm^−1^ band of calcite at lower spectral resolution and characterize the samples of wood ash. The system incorporates an Olympus BX51 microscope and the 50 × objective was used. Analyses were conducted with a laser power of 1.0 mW in 35 scans. The spectrometer worked in the 3350–55 cm^−1^ spectral range. Spectra were processed with OMNIC v. 8.2 using the Gaussian/Lorentzian function and polynomial baseline.

## Data Availability

The research data on which this publication is based are available from the authors upon request. MBT should be contacted for FTIR data; for Raman micro-spectroscopy data, IP (1800 gr/mm measurements) and AAG (900 gr/mm).
